# A Corticotropin-Secreting Adenoma in the Setting of von Hippel-Lindau Disease

**DOI:** 10.1210/jcemcr/luaf055

**Published:** 2025-04-10

**Authors:** Xiaoxue Chen, Yue Zhou, Lin Lu, Ming Feng, Linjie Wang, Anli Tong

**Affiliations:** Key Laboratory of Endocrinology of National Health Commission, Department of Endocrinology, Peking Union Medical College Hospital, Chinese Academy of Medical Science and Peking Union Medical College, Beijing 100730, China; Key Laboratory of Endocrinology of National Health Commission, Department of Endocrinology, Peking Union Medical College Hospital, Chinese Academy of Medical Science and Peking Union Medical College, Beijing 100730, China; Key Laboratory of Endocrinology of National Health Commission, Department of Endocrinology, Peking Union Medical College Hospital, Chinese Academy of Medical Science and Peking Union Medical College, Beijing 100730, China; Department of Neurosurgery, Peking Union Medical College Hospital, Chinese Academy of Medical Sciences, Beijing 100730, China; Key Laboratory of Endocrinology of National Health Commission, Department of Endocrinology, Peking Union Medical College Hospital, Chinese Academy of Medical Science and Peking Union Medical College, Beijing 100730, China; Key Laboratory of Endocrinology of National Health Commission, Department of Endocrinology, Peking Union Medical College Hospital, Chinese Academy of Medical Science and Peking Union Medical College, Beijing 100730, China

**Keywords:** von Hippel-Lindau (VHL) disease, adrenocorticotropic hormone (ACTH), ACTH-secreting adenoma, pituitary adenoma

## Abstract

Von Hippel-Lindau (VHL) disease is an autosomal dominant disorder caused by germline pathogenic variants of the *VHL* gene, which can lead to abnormal growth of blood vessels and cause the development of benign or malignant tumors, as well as cysts in diverse organs. To date, no case reports have documented adrenocorticotropic hormone (ACTH)-secreting adenomas in individuals with VHL disease. We present the case of a 19-year-old female individual with VHL disease who developed an ACTH-secreting adenoma alongside hemangioblastomas in the central nervous system (CNS) and cystic lesions in diverse organ systems. Genetic testing and immunohistochemistry of the pituitary tumor were performed. Genetic testing revealed that the patient carried the familial germline pathogenic variant located in the first exon of the *VHL* gene (c.227_229del, p.76delF). Immunohistochemical staining of the pituitary tumor demonstrated positive for ACTH, chromogranin A, and synaptophysin, with Ki-67 index at 3%. In addition, tumor cells showed scattered immunoreactivity for the α subunit of hypoxia-inducible factor (HIF-1α). This case suggests that VHL disease might be associated with ACTH-secreting adenomas and broadens the tumor spectrum.

## Introduction

Von Hippel-Lindau (VHL) disease (MIM 193300) is an autosomal dominant disorder caused by germline pathogenic variants of the *VHL* gene, located on the short arm of chromosome 3 [1]. This condition has an estimated global prevalence of approximately 1 in 35 000 individuals [1]. These variants disrupt normal angiogenesis, leading to the development of benign or malignant tumors, and cysts in diverse organs. Retinal and cerebellar hemangiomas are the most prevalent tumors, along with spinal cord hemangioblastoma, renal cell carcinoma, pheochromocytoma, and pancreatic tumors [1].

The *VHL* gene encodes a tumor-suppressor product (pVHL), which plays a critical role in various cellular processes, including the proteasome degradation, the cilia integrity maintenance, and the extracellular matrix regulation. pVHL targets the α subunits of hypoxia-inducible factor (HIF) 1 and 2. In its absence, HIF-1 and HIF-2 may stimulate tumor growth by upregulating the expression of vascular endothelial growth factor (VEGF), and other growth factors [2].

Although pituitary adenomas have been reported in association with VHL disease, including a null cell pituitary tumor and a growth hormone-prolactin (GH-PRL) pituitary adenoma, no cases of VHL disease with Cushing disease have been described to date [3, 4]. Here, we present the first case of an adult with VHL disease who developed an adrenocorticotropic hormone (ACTH)-secreting pituitary adenoma.

## Case Presentation

A 19-year-old female patient was referred to our hospital due to the facial fullness and purple striae for about 2 years. At the age of 17, she gradually developed a round and red face with facial acne. At the same time, she exhibited typical central obesity, accompanied by wide purple striae on the abdomen. She gained about 15 kilograms in a year, with symptoms of fatigue and hair loss. Several months later, she began to suffer from dryness of mouth and polydipsia, consuming about 2000 to 2500 mL of water daily. She had a regular menstrual cycle, with a cycle length of approximately 30 days, and each menstrual period lasted about 5 days. During a hospitalization for a vulvar abscess, her random blood glucose was found to be 414 mg/dL (23 mmol/L) (normal reference range: 70-200 mg/dL; 3.9-11.1 mmol/L) and urinary ketone bodies were strongly positive. Laboratory tests also revealed hypokalemia and hypernatremia: serum potassium 9.9 mg/dL (2.55 mmol/L) (normal reference range: 13.7-21.5 mg/dL; 3.5-5.5 mmol/L), serum sodium 338.3 mg/dL (147.1 mmol/L) (normal reference range: 310.5-333.5 mg/dL; 135-145 mmol/L). The plasma cortisol was 59.9 μg/dL (1655 nmol/L) at 8 Am (normal reference range: 5-25 μg/dL; 138-690 nmol/L), 40.4 μg/dL (1117 nmol/L) at 4 Pm and 31.8 μg/dL (879.8 nmol/L) at midnight, indicating excessive cortisol and disturbed rhythm. ACTH levels were unsuppressed, and a low-dose (1 mg) overnight dexamethasone suppression test failed to suppress cortisol (41.2 μg/dL; 11 394 nmol/L). She also had hypertension, peaking at 160/120 mmHg. The patient was treated with insulin, potassium chloride oral solution, antihypertensive drugs, and antibiotics in the local hospital. After treatment, her fasting blood glucose decreased to between 126 and 144 mg/dL (7-8 mmol/L), blood pressure stabilized below 140/90 mmHg, and other symptoms improved. The serum potassium and sodium levels of the patient gradually returned to normal. However, no further assessment of hypercortisolemia was performed.

**Figure 1.**
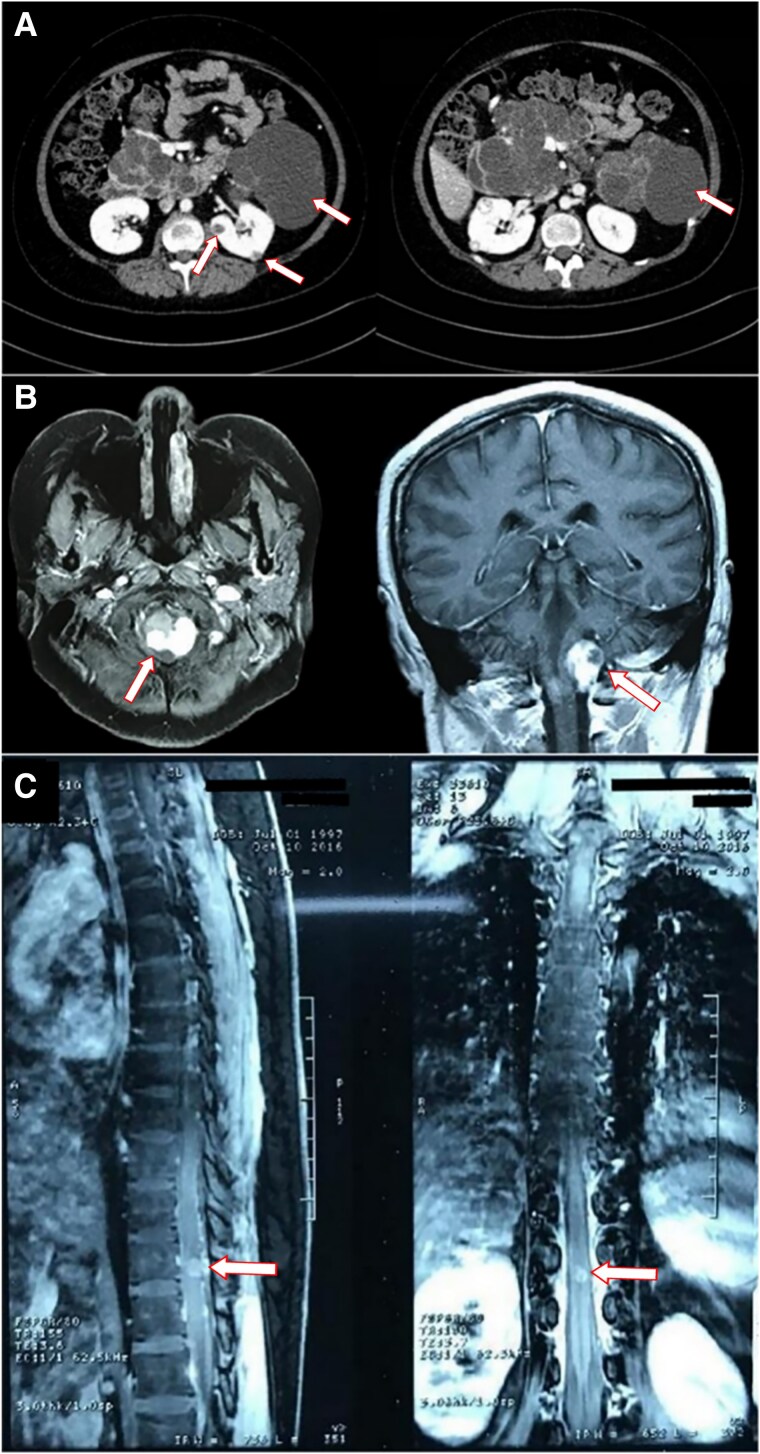
Some results of imaging examination. A, The abdominal enhanced computerized tomography (CT). The arrows show the cysts in both kidneys and pancreas. B, The magnetic resonance imaging (MRI) of the head. The arrows show the lesion in bilateral cerebellar bulbar cisterna. C, The magnetic resonance imaging (MRI) of the thoracic vertebrae. The arrows show the lesion in the thoracic vertebrae.

One month later, she suddenly experienced leg numbness and tingling, unable to stand or walk. Subsequently, she felt a sensation of formication and burning on her abdomen, accompanied by lower back pain. She visited the outpatient service of a local hospital. Bone mineral density examination suggested osteoporosis, primarily characterized by reduced bone mass in the lumbar spine. Abdominal enhanced computerized tomography (CT) showed the presence of cysts in both kidneys and pancreas, along with pancreatic duct dilatation (Fig. 1A). In addition, magnetic resonance imaging (MRI) of head revealed multiple solid lesions in bilateral cerebellar bulbar cisterna (Fig. 1B) and mass within the left eyeball. MRI of thoracic vertebrae indicated spinal vascular malformation and hemorrhage within the spinal cord, with a high probability of cavernous hemangioma (Fig. 1C). She underwent operations to remove the tumors in the spinal cord and the medulla oblongata. Histopathological examination confirmed the diagnosis of hemangioblastomas. She was subsequently referred to our hospital for evaluation of hypercortisolemia.

The family history was notable for suspicious tumors in her mother, maternal uncle, and maternal grandfather. Her maternal grandfather died of kidney cancer, and her maternal uncle died at age 21 of lower back pain and lower limb paralysis. Additionally, her mother had pancreatic cysts and diabetes, which was diagnosed at 39.

## Diagnostic Assessment

**Figure 2.**
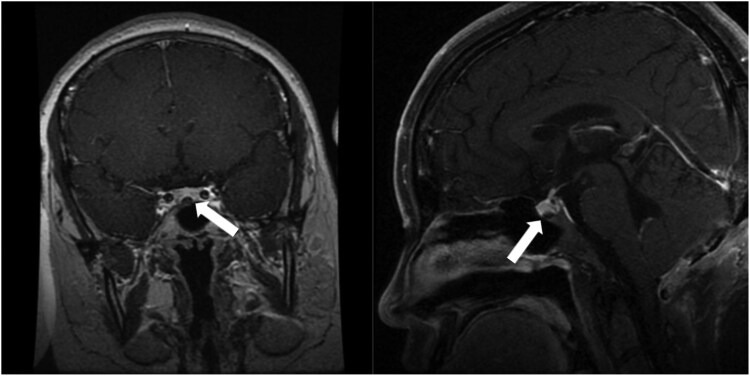
The magnetic resonance imaging (MRI) of the pituitary. The arrows show a decreased enhancement area in the midline area of the lower pituitary, with the possibility of microadenoma.

The patient's cortisol levels were measured multiple times, revealing a loss of the normal circadian rhythm. Her baseline serum cortisol level was 26.8 μg/dL (742 nmol/L) and 24-hour urinary cortisol was 359.85 μg/24 hours (9946 nmol/24 hours) (normal reference range: 12.3-103.5 μg/24 hours; 340-2861 nmol/L). A low-dose dexamethasone suppression test failed to suppress cortisol: her morning plasma cortisol was 25.5 μg/dL (704 nmol/L), 24-hour urine cortisol 394.6 μg/24 hours (10 906 nmol/24 hours). Her ACTH concentration was 36.2 pg/mL (8 pmol/L) (normal reference range: 7.2-63.3 pg/mL; 1.6-14 pmol/L), highly suggestive of ACTH-dependent Cushing syndrome. After a high-dose dexamethasone suppression test, the serum cortisol was 2.4 μg/dL (61.9 nmol/L) and 24-hour urine cortisol was 163.4 μg/24 hours (4517 nmol/L), suggesting cortisol level was suppressed. Subsequently, the dynamic enhanced MRI of the pituitary revealed a decreased enhancement area (5.54 mm × 3.6 mm) in the midline area of the lower pituitary, with the possibility of microadenoma (Fig. 2). To further elucidate the etiology, we conducted inferior petrosal sinus sampling with desmopressin for ACTH stimulation, which revealed that the disease was due to ACTH-secreting tumor of the pituitary gland rather than an ectopic lesion (Table 1). These findings established a definitive diagnosis of Cushing disease. In addition, the patient's other anterior pituitary hormones were within normal ranges.

**Table 1.** Adrenocorticotropic hormone levels of inferior petrosal sinus venous blood and peripheral venous blood

Minutes	Right inferior petrosal sinus	Left inferior petrosal sinus	Peripheral blood
0	39.1 pg/mL (8.6 pmol/L)	119 pg/mL (26.2 pmol/L)	17.5 pg/mL (3.8 pmol/L)
3	235 pg/mL (51.6 pmol/L)	>1250 pg/mL (>274.7 pmol/L)	19.8 pg/mL (4.4 pmol/L)
5	445 pg/mL (97.8 pmol/L)	>1250 pg/mL (>274.7 pmol/L)	31.4 pg/mL (6.9 pmol/L)
10	475 pg/mL (104.4 pmol/L)	>1250 pg/mL (>274.7 pmol/L)	85.4 pg/mL (18.8 pmol/L)

Normal reference range for peripheral blood sampling: 7.2-63.3 pg/mL, 1.6-14 pmol/L. Reference range for inferior petrosal sinus sample: if the ratio of adrenocorticotropic hormone concentration in the inferior petrosal sinus to that in the peripheral blood is greater than 3, it indicates the source of adrenocorticotropic hormone is the pituitary gland rather than an ectopic lesion.

**Figure 3.**
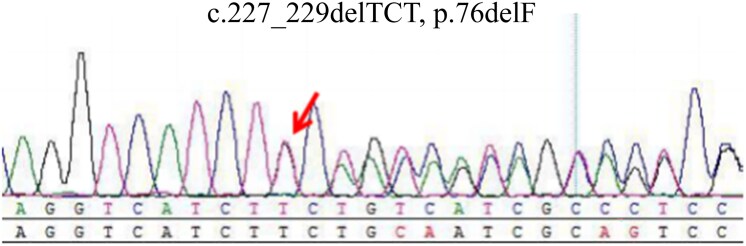
The genetic testing result of the patient. The arrow shows the location of the mutation.

Genetic testing identified a familial germline pathogenic variant in exon 1 of the *VHL* gene (c.227_229del, p.76delF) (Fig. 3).

## Treatment

**Figure 4.**
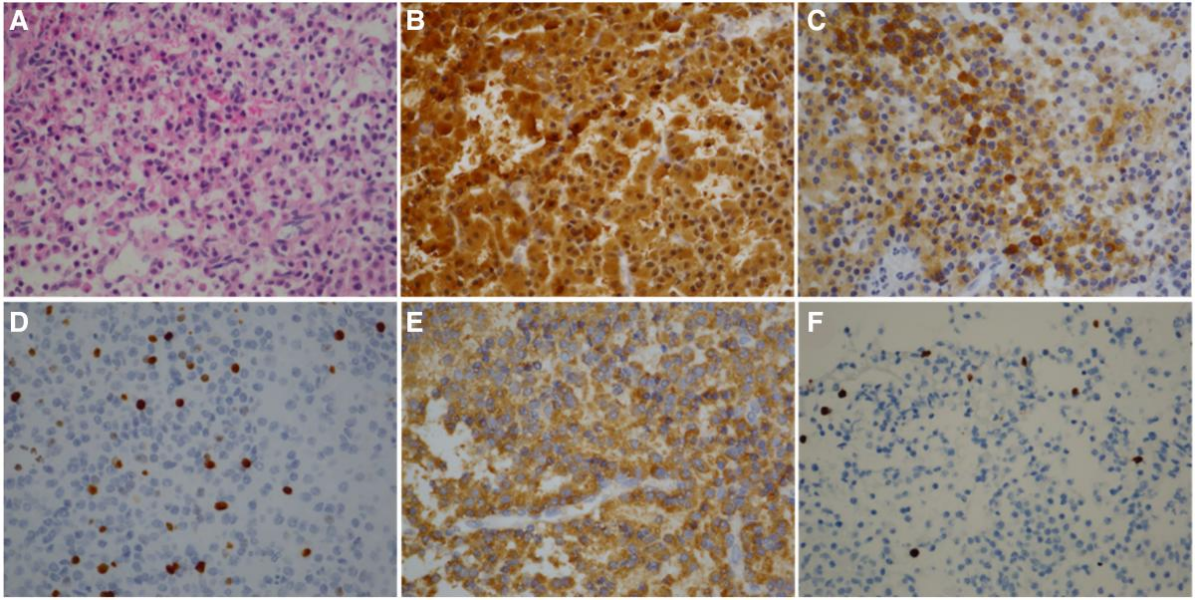
Characteristics of tumor cells. A, Hematoxylin and eosin stain. B-F, Immunohistochemical staining for ACTH (B), chromogranin A (C), Ki-67 index (D), growth hormone (E), and HIF-1α (F).

The sellar tumor was removed through a transsphenoidal approach. Immunohistochemical staining was positive for ACTH, chromogranin A, and synaptophysin, with Ki-67 index at 3% (Fig. 4A-4D). The tumor cells exhibited partial positivity for growth hormone (Fig. 4E), but were negative for prolactin, thyroid stimulating hormone, follicle-stimulating hormone, and luteinizing hormone. In addition, tumor cells showed scattered immunohistochemical staining for HIF-1α (Fig. 4F).

## Outcome and Follow-Up

On the first day after surgery, the patient's ACTH concentration at 8 Am was 17.5 pg/mL (3.9 pmol/L) and serum cortisol level was 5.49 μg/dL (151.7 nmol/L). Three days later, the concentration of ACTH dropped to less than 5 pg/mL (1.1 pmol/L), and the serum cortisol declined to 0.66 μg/dL (18.2 nmol/L). No other symptoms were observed. The patient recovered well and was discharged on the fifth day after the surgery. At discharge, she was advised to continue pharmacological management for blood sugar and blood pressure control and to attend a follow-up visit at the outpatient department 1 month later. However, the patient did not return for the scheduled follow-up, precluding further assessment of the therapeutic outcomes.

## Discussion

VHL disease results from germline pathogenic variants in the *VHL* tumor-suppressor gene on chromosome 3p25-p26 [5]. Patients with VHL disease may develop lesions in various organs and the central nervous system (CNS), including renal cell carcinoma or cysts, pheochromocytoma, pancreatic neuroendocrine tumors or cysts, benign cystadenomas of adnexal organs, and CNS hemangioblastomas [6]. To date, only a few cases of pituitary adenomas have been reported in individuals with VHL disease, including a GH-PRL adenoma and a null cell adenoma (Table 2) [3, 4]. However, to our knowledge, ACTH-secreting adenomas have not been previously described in VHL disease, although ectopic Cushing syndrome due to pancreatic neuroendocrine tumors has been described in 2 young female individuals [7, 8]. Here, we report the first case of a patient with an ACTH-secreting adenoma in the setting of VHL disease who presented with Cushing disease during adolescence.

**Table 2.** Reported cases of pituitary adenomas in the setting of von Hippel-Lindau disease

Author (year)	Age (yrs)/Sex	Family history	VHL-associated visceral tumor	VHL-associated CNS tumor	Histology of pituitary adenomas	Gene study
Tudorancea et al [3]	18/M	Mother: cerebellar/retinal hemangioblastomas, renal tumor, pancreatic cysts. Maternal aunt: cerebellar/cerebral trunk/retinal hemangioblastomas. Maternal grandmother: cerebellar/retinal hemangioblastomas.	No	Retinal vascular lesions/cerebellar hemangioblastomas	Growth hormone-prolactin-secreting adenoma	Familial germline pathogenic variant located in the first exon of *VHL* gene (c.340G>C, p.Gly114Arg)
Shimoda et al [4]	73/M	No	No	Cerebellar hemangioblastomas	Null cell adenoma	NA
Present case	19/F	Mother: pancreatic cysts. Maternal grandfather: kidney cancer. Maternal uncle: lower limb paralysis.	Pancreatic/renal cysts	Medulla oblongata/spinal cord/retinal hemangioblastomas	Corticotropin-secreting adenoma	Familial germline pathogenic variant located in the first exon of *VHL* gene (c.227_229del, p.76delF)

Abbreviations: CNS, central nervous system; F, female; M, male; NA, not available; VHL, von Hippel-Lindau.

Genetic testing, including sequencing of the *VHL* gene and copy number variation analysis identified a pathogenic deletion mutation at nucleotide 228 in exon 1. This variant was pathogenic or likely pathogenic according to ClinVar (https://www.ncbi.nlm.nih.gov/clinvar/RCV000208790/). This finding aligned with previous reports of a family with a deletion mutation at nucleotide 227 in exon 1, in which affected individuals exhibited renal lesions requiring surgical intervention [9]. Similarly, the patient in this case also presented with multiple renal cysts.

In VHL disease, pathogenic variants disrupt pVHL synthesis, impairing HIFα degradation and mimicking hypoxia. This triggers transcription of hypoxia-related genes like VEGF, causing abnormal vascular hyperplasia and clinical manifestations [10]. pVHL is widely expressed, particularly in nervous systems and VHL-affected organs [11]. Interestingly, pituitary eosinophils exhibited pVHL immunoreactivity, although the pituitary gland was rarely affected in VHL disease [11]. Immunohistochemical staining indicated the cytoplasmic expression of pVHL in most adenohypophyseal cells, with 53 of 68 pituitary adenomas showing pVHL expression—32 cytoplasmic, 7 nuclear, and 14 both [12]. Notably, growth hormone tumors with less vascularization predominantly exhibited nuclear pVHL localization, suggesting a potential role in inhibiting pituitary angiogenesis, as transcriptional regulation tended to be localized in the nucleus.

Tumor neovascularization and vascular remodeling are critical for pituitary adenoma growth and progression [13]. Studies of 30 hormone-negative pituitary adenomas revealed upregulated VEGF expression in low pVHL groups, correlating with higher recurrence rates [14]. Immunohistochemistry of HIF-1α in pituitary tumors showed scattered expression in tumor cells and microvascular endothelial cells, but there was heterogeneity among different subtypes, with the weakest expression in ACTH tumors [15]. Notably, the pituitary tumor we reported was immunoreactive for HIF-1α, which might be associated with *VHL* pathogenic variants. HIF-1α enhances the expression of VEGF-A, stabilized by the RWD-containing sumoylation enhancer (RSUME) [16]. Invasive pituitary adenomas showed higher RSUME, HIF-1α, and VEGF-A levels than non-invasive ones, and the expression of RSUME was positively correlated with HIF-1α/VEGF pathway activity [17]. Although clinical cases are limited, *VHL* germline pathogenic variants may contribute to the development and aggressive behavior of pituitary tumors through vascular abnormalities.

The patient experienced the typical comorbidities of Cushing disease, including hypertension, glucose metabolism impairment, bone disease, and infections [18]. Diagnosis of Cushing disease is frequently delayed for several years, partly due to insufficient awareness of the insidious and progressive nature of the disease, as well as the complexity involved in testing [19, 20]. Such delays in initiating diagnosis may result in severe consequences, including hypercoagulability, hypertension, and increased susceptibility to infections, which are particularly dangerous for patients with VHL disease who may have cysts and hemangiomas in the organ. Therefore, timely diagnosis is critical for these patients.

In conclusion, this case report expands the clinical spectrum of VHL disease by highlighting ACTH-secreting adenomas as a potential manifestation. Although only 2 previous cases of VHL-associated pituitary tumors have been reported, future cases may reveal additional pituitary adenoma subtypes as part of the disease phenotype.

## Learning Points

- Pituitary adenomas rarely occur in the setting of von Hippel-Lindau disease, but isolated cases have been reported.
- Despite the patient's VHL background, her symptoms of Cushing disease were classic, and the diagnostic and therapeutic approach adhered strictly to established guidelines. This case underscores the importance of maintaining proficiency in diagnosing and managing common conditions, even when treating rare and complex diseases.
- Clinicians should remain vigilant for rare comorbid patterns and investigate potential underlying connections between coexisting diseases.

## Contributors

All authors made individual contributions to authorship. X.C. collected and interpreted the data and wrote the manuscript. Y.Z. and L.L. were involved in the diagnosis and management of the patient. M.F. was responsible for the patient's surgeries. L.W. and A.T. revised the manuscript and provided funding support. All authors reviewed and approved the final draft.

## Data Availability

Original data generated and analyzed during this study are included in this published article.

## Abbreviations

ACTH: adrenocorticotropic hormone
CNS: central nervous system
GH-PRL: growth hormone-prolactin
HIF: hypoxia-inducible factor
MRI: magnetic resonance imaging
pVHL: Von Hippel-Lindau tumor-suppressor protein
RSUME: RWD-containing sumoylation enhancer
VEGF: vascular endothelial growth factor
VHL: Von Hippel-Lindau
